# Collaborative encoding with a new categorization task: a contribution to collaborative memory research

**DOI:** 10.1007/s00426-024-01929-w

**Published:** 2024-03-11

**Authors:** Nadia Conte, Santiago Pelegrina, Caterina Padulo, Erika Marascia, Beth Fairfield

**Affiliations:** 1 Department of Psychological, Health and Territory Sciences, G. D’Annunzio University of Chieti, 66100 Chieti, Italy; 2 Department of Psychology, University of Jaén, 23071 Jaén, Spain; 3 Department of Humanities, University of Naples Federico II, 80133 Naples, Italy

## Abstract

Collaborative memory research has focused primarily on the effects of collaboration at recall with collaboration during encoding receiving less attention. In the present study, collaboration was investigated both at encoding and at retrieval to determine its effects and possible interactions. The aim was to clarify whether the collaborative inhibition effect depended on whether the encoding was with the same or with a different partner. A total of 320 participants (160 Italian and 160 Spanish undergraduate students) were administered a modified version of the collaborative memory paradigm with a new categorization task of verbal affective stimuli at encoding. Specifically, they were asked to classify 90 printed words into 6 categories, so as to have 15 words in each category and then did two recall tasks. Participants were assigned to one of five possible conditions according to encoding (collaborative, individual) and recall (collaborative with the same partner, with another partner, and individual). Results show a collaborative encoding deficit and the classic collaborative inhibition effect independently of whether the encoding was collaborative or individual and even in a collaborative recall group that showed a degree of recall output organization comparable to that of the individual recall group. These results are not wholly consistent with a retrieval disruption explanation and are discussed according to divided attention during collaborative recall and how it may contribute to the collaborative inhibition effect.

**Supplementary Information:**

The online version contains supplementary material available at 10.1007/s00426-024-01929-w.

## Introduction

Human memory has typically been studied in laboratory settings in which individuals encode and remember alone (e.g., Miller, 1956; Baddeley et al., 1975; Cowan 2010). Yet, innumerous studies have shown how memory is influenced by surrounding social contexts (Wyer & Srull, 2014). Indeed, individuals are social beings, situated in precise socio-cultural contexts within which they continuously interact with other people (Read & Miller, 1995). Therefore, laboratory studies in which social interactions are recreated are necessary to further understanding of human memory and how it is shaped by collaboration both during the encoding and recall of information.

### Collaborative memory

Results from studies on collaborative memory in which individuals encode information and then remember together in a first recall and individually in a second recall, generally show that after the initial individual encoding phase, people who collaboratively recalled items (e.g., in dyads or triads) remembered less non-redundant information than the same number of people recalling individually. Weldon and Bellinger (1997) termed this phenomenon, collaborative inhibition.^1^

Various explanations have been advanced to explain these results. For example, social loafing, the tendency for individuals to put forth less effort when they are part of a group, has long been considered the most obvious explanation, but it seems that collaborative inhibition does not occur due to the diffusion of responsibility (Weldon et al., 2000). Rather, results seem to suggest that collaborative inhibition is a cognitive phenomenon more similar to the part-set cueing deficit, a recall impairment after participants are given some studied items as retrieval cues (Basden et al., 1997; Nickerson, 1984). This outcome may be similar to what emerges with the socially shared retrieval-induced forgetting effect. When listeners attend to the speakers’ selective recall of previously encoded information, they forget unmentioned but related information (Coman & Hirst, 2015; Cuc et al., 2007). Although these effects might be related to an inhibitory mechanism, subsequently, retrieval competition was proposed (Rundus, 1973), but evidence shows that the strengthening of a subset of elements does not necessarily lead to the forgetting of other elements (Bäuml & Aslan, 2004). Considerable evidence supports the retrieval disruption hypothesis (Basden et al., 1997). This hypothesis posits that individuals develop their own idiosyncratic organization of information while encoding and that later, during recall, this organizational structure is disrupted when information is retrieved in a different order by another participant. This result leads to a decrease in recall.

### Collaborative encoding

Most collaborative memory studies have focused primarily on collaborative recall. Yet, daily life is full of situations in which people commonly engage in collaborative "encoding”. For example, Brown (2003) found that students who collaboratively generated conceptual maps outperformed students who worked alone. It has been proposed that collaborative encoding may counteract the effect of collaborative inhibition because people who collaboratively encode information develop more compatible retrieval strategies thus reducing the potential interference during retrieval. Nonetheless, only a few studies have investigated collaborative encoding and those have found a heterogeneity in results.

Andersson and Rönnberg (1995) tested the effects of several factors on collaboration and observed no collaborative encoding benefits. Specifically, in Experiment 2, subjects watched a 34-min videotape without talking to each other and after a 2-min delay, were asked to discuss the video. Following discussion, subjects performed a first individual recall and a second individual or collaborative recall. Results showed stronger collaborative inhibition in the participants who discussed the video compared to those who did not. As participants who collaborated showed a recall impairment, researchers interpreted findings as support for collaborative encoding deficit. However, in their study, the encoding task was not an active task and may have been unsuccessful in creating similar idiosyncratic cognitive organizational structures. On the contrary, other studies have shown that collaborative encoding eliminates the collaborative inhibition effect (Barber et al., 2012; Finlay et al., 2000; Harris, Barnier and Sutton, 2013). Thus, Finlay et al., (2000) manipulated collaboration both at encoding and recall. In this study, the encoding task was designed to align the sequential organization of stimuli and this type of collaborative encoding led partners to adopt similar organizational patterns. Results showed that collaborative inhibition decreased or was eliminated when partners adopted a similar retrieval strategy, in line with the retrieval strategy disruption hypothesis. The study by Harris, Barnier, and Sutton (2013) also provided evidence that collaborative encoding eliminates the collaborative inhibition effect when involved in active collaboration. It should be noted that if the collaborative inhibition effect is determined by the disruption of recall strategies, collaboration with the same partner during both encoding and retrieval phases should reduce the disruption and consequently mitigate this negative effect; whereas recall with a different partner should not counteract the collaborative inhibition effect. The study by Barber et al. (2010) explored this possibility but did not yield conclusive results. They observed similar recall performance among participants who collaborated during encoding, regardless of whether the subsequent recall was performed individually, with the same partner, or with a different partner than the one in the study phase. As the collaborative inhibition effect was also absent, the interesting question of whether the reduction of the effect depends on the partner remains unresolved.

Collaborative encoding, in addition to its potential influence on the collaborative inhibition effect, may also have an overall negative effect on recall performance. It is also possible that collaboration during encoding influences subsequent recall, not only via the similarity of retrieval strategies or the compatibility of retrieval cues but by directly affecting the degree of learning of the material. Collaborative encoding may divert attention away from the encoding task itself (Barber et al., 2012). Several studies have demonstrated the inextricable link between attention and memory and have shown how divided attention (DA) can affect memory performance (Anderson et al., 1998; Baddeley et al., 1984; Craik et al., 1996). Specifically, DA during encoding negatively affects both memory and secondary task performance, compared to full attention (FA) conditions (Fernandes & Moscovitch, 2000; Naveh-Benjamin et al., 1998, 2000). Naveh-Benjamin et al. (2000) argued that DA at encoding changes the quality of encoding that shifts from a more semantically elaborated to a more superficial type. Other studies have also shown that DA during encoding has disruptive effects on recollection-based processing (Yonelinas, 2002), affecting the retrieval of contextual information associated with the target (Sacher et al., 2009).

So far, few studies have focused on collaborative encoding and none of these has shown that collaborating during encoding is beneficial for recall. Some studies have reported that at least under certain circumstances, collaboration during encoding may counteract the negative collaborative-inhibition effect that occurs during the later recall phase. This could depend on whether the recall is individual or in collaboration with the same encoding partner or with a different one, although there is currently no conclusive evidence to support this hypothesis. Therefore, it is important to investigate how collaboration both during encoding and recall influences recall performance.

### Classical collaborative memory paradigm with emotional stimuli

The collaborative memory paradigm has also been used with a variety of stimuli with emotional valence (e.g., positive, neutral, and negative stimuli) including pictures (Barber et al., 2017), movies (Andersson & Rönnberg, 1995), events (Yaron-Antar & Nachson, 2006), and short videos (Wessel et al., 2015). In studies that used emotional stimuli, the classic effects of collaborative inhibition (Vredeveldt et al., 2016; Wessel et al., 2015; Yaron-Antar et al., 2006) and individual post-collaborative benefits (Bärthel et al., 2017; Yaron-Antar & Nachson, 2006) were generally confirmed.

While it is important to investigate the mechanisms of collaborative recall and the influences that the use of emotional stimuli may have on recall performance, it is equally important to consider that when remembering, we not only remember information collaboratively, but we may also encode it collaboratively. In most of the studies on collaborative memory, including those that have used emotionally valanced stimuli, the encoding phase was individual. Collaborative encoding with valanced material remains to be explored.

### The present study

Collaboration during encoding may induce detrimental and beneficial effects on recall performance. That is, collaborative encoding deficits and collaborative inhibition effects. In the present study, we aimed to comprehensively examine both effects and to investigate how the composition of collaborative groups (involving the same or different partners during the encoding and recall phases) affects recall performance and patterns of organization of recalled information. Based on the retrieval disruption hypothesis (Basden et al., 1997; see Rajaram & Pereira-Pasarin for a review), the reduction of the collaborative inhibition effect may depend on how the collaborative groups are configured during recall: whether they are formed by the same or different partners who encoded the material. Groups with the same partners in both phases may develop similar retrieval strategies, which in turn contributes to reducing the collaborative inhibition effect. To this end, we developed a task that requires an active collaboration among participants allowing us to determine the extent to which similar organizational strategies are used by partners in different collaborative groups.

Some studies may not effectively produce collaborative effects due to the conditions and tasks employed. For example, tasks like the one used in the study by Andersson and Rönnberg (1995) may not facilitate active collaboration among participants. To address this, we developed a modified version of the collaborative memory paradigm to include an encoding task that stimulates discussion and active collaboration among participants, indispensable aspects for effective collaborative encoding. The task involved the organization of the material to be studied (Basden et al., 1997, 2000). Specifically, we used a categorization task where participants, either individually or in pairs, classified affective word stimuli into provided categories. While the categories of some words were clear (e.g., HEALTH-nurse), others were intentionally ambiguous, requiring participants to reach a consensus through discussion. In addition, whereas prior collaborative encoding studies relied on recall performance to infer the effects of organizational similarity on performance, we introduced an objective measure of organization to more directly examine whether recall output organization was compatible with the organization established during encoding. Specifically, we used the adjusted ratio of clustering (ARC—Roenker et al., 1971) to assess organization at recall and as an index of the degree of disruption of the retrieval strategies (Basden et al., 1997).

**Fig. 1:**
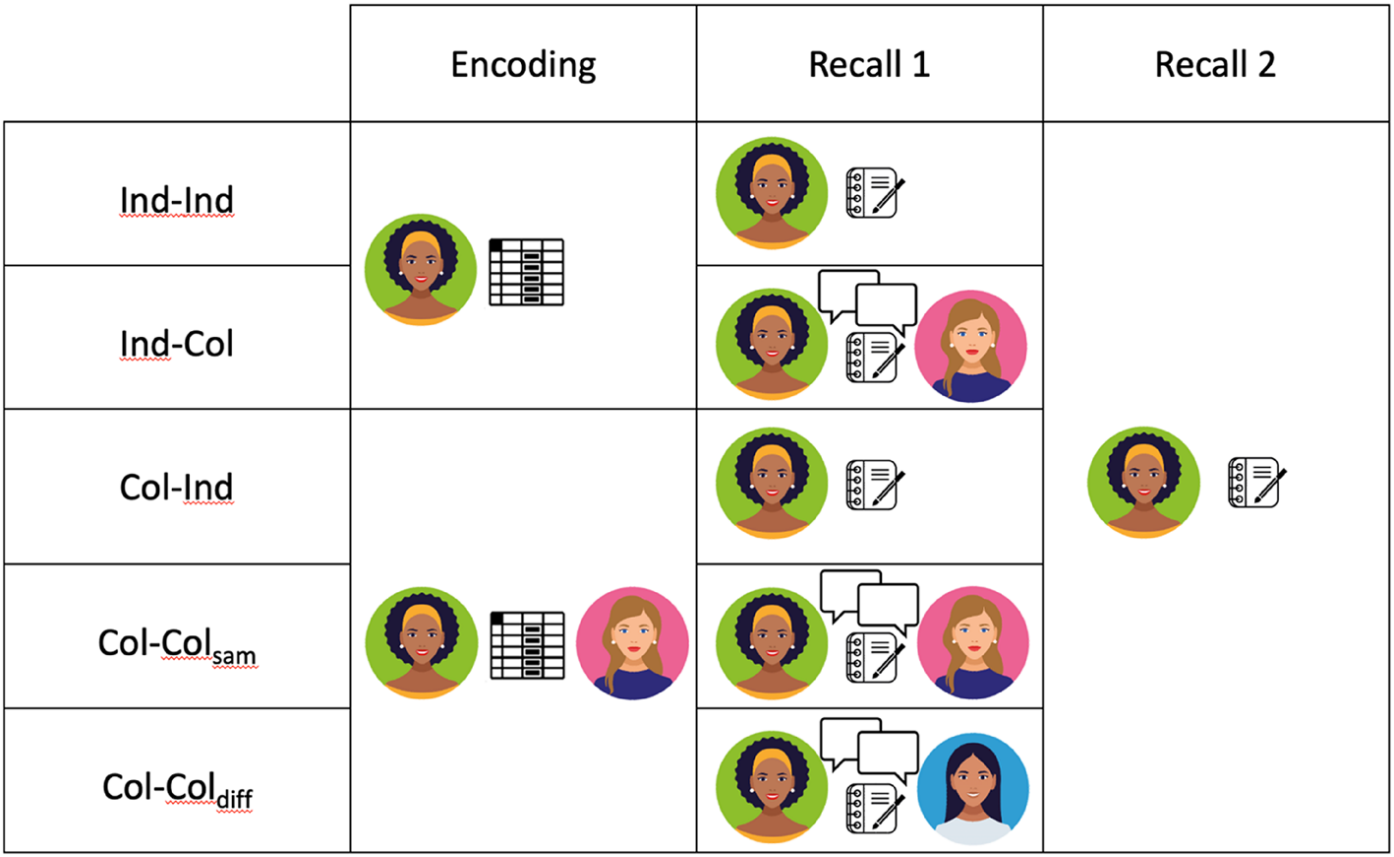
Outline of the study design indicating the five conditions and the three principal phases. *Note.* Ind–Ind: individual encoding and individual recall, Ind–Col: individual encoding and collaborative recall, Col–Ind: collaborative encoding and individual recall, Col–Col_diff_, collaborative encoding and collaborative recall with a different partner, and Col–Col_sam_: collaborative encoding and collaborative recall with the same partner. Images adapted from: https://it.vecteezy.com

Furthermore, here, we expanded our investigation by including not only the four conditions that arise from the factorial combination of encoding and recall types (individual or collaborative), critical for assessing the intended effects; but also, by adding an extra condition introduced in Barber's (2010) study consisting of collaborative recall with a different partner than during the encoding phase. This condition allows us to investigate whether the elimination or reduction of the collaborative inhibition effect requires having the same partner in the recall phase as in the collaborative encoding phase. Thus, the following conditions were included: in the Ind–Ind condition participants individually encoded and recalled individually; in the Ind–Col condition encoded individually and recalled dyadically; in the Col–Ind condition encoded dyadically and recalled individually; in the Col–Col_sam_ condition encoded dyadically and recalled dyadically with the same partner, and in the Col–Col_diff_ condition encoded dyadically and recalled dyadically with another partner. Finally, we included an additional individual recall phase, to determine whether possible effects (collaborative inhibition, encoding deficit) obtained in the first recall were transitory or long-lasting. Figure 1 depicts the study design and the task phases.

Considering the relevant research reviewed, we hypothesized that collaborative encoding hinders recall, since encoding takes place under divided attention. In contrast, when encoding is individual, items may receive full attention and memory should increase. Thus, our first hypothesis predicts that collaborative encoding deficits on both the number of items remembered and ARC scores. In line with this, we expected better recall following individual than collaborative encoding, regardless of recall condition (individual or collaborative). Moreover, if the collaborative encoding deficit is due to poor learning of the material, this effect should be long-lasting and observed also on the second individual recall trial.

The second hypothesis predicts a collaborative inhibition effect, whereby collaborative recall generates negative effects on nominal recall, specifically when collaboration occurs with a different partner than the original encoding partner (i.e., Col–Col_diff_) in line with the retrieval disruption hypothesis (Basden et al., 1997). It should be evident in ARC scores as they reflect the organization of the studied information, as well as on nominal recall. We also expected (third hypothesis) a reduction (or even an elimination) of the collaborative inhibition effect when the same partners collaborate at encoding and recall. This reduction should be driven by the similar organization of the material and cross-cuing, reducing episodes that disrupt the retrieval processes of a member group. We expected to observe it in both nominal recall and ARC scores since it depends on the organization of the material. Finally, the fourth hypothesis posits that among groups that encoded collaboratively, those who do not experience retrieval disruption (Col–Ind or Col–Col_sam_) should perform better than those who experienced retrieval disruption when recalling collaboratively with a different partner (Col–Col_dif_). Furthermore, if the collaborative inhibition effect is due to disruption strategies resulting from the collaboration between partners with different organizations of information, this detrimental effect should not be observed in a second individual recall in which such strategy disruption is absent.

## Methods

### Participants

The sample size was based on prior research investigating collaborative inhibition and collaborative encoding deficit (Barber et al., 2010; Harris et al., 2013) that included 48–69 participants by condition. A total of 320 participants completed the tasks. 160 Italian (age: *M:* 20.46, *SD:* 3.19; education: *M*: 14.80, *SD*: 1.23) undergraduate students from the University of Chieti (Italy) and 160 Spanish (age: *M:* 19.81, *SD:* 3.09; education: *M*: 14.01, *SD*: 1.05) undergraduate students from the University of Jaén (Spain) were recruited. Utilizing two samples from different countries enables us to assess the generalizability of the results across both populations. Participants were assigned to one of five possible conditions before beginning the study. Informed consent was obtained from all participants, following the Helsinki protocol (World Medical Association, 2013). The present study was approved by the Ethical Committee of the University of Jaén (prot. NOV.21/2.PRY).

### Stimuli

A total of 90 affective words (30 neutral, 30 negatives, and 30 positives) were selected from the Italian (Montefinese et al., 2014) and the Spanish (Redondo et al., 2007) versions of the ANEW database (Bradley & Lang, 1999) for the Italian and the Spanish administration respectively.

We selected six general categories: Health, Environment, News, Behavior, Education, Relationships, so that the word stimuli were in some cases easily categorizable (e.g., the word "*nurse*" intuitively will go into the HEALTH category) and in other cases difficult to categorize (e.g., "*hate*" which could fit into either RELATIONSHIPS or BEHAVIORS categories). In general, all words differed in valence (*p* < 0.001). Neutral words differed from negatives and positives (*p* < 0.001) in arousal, but negatives and positives did not differ in arousal (*p* > 0.26); all words were comparable for length, frequency of use, and familiarity (*p* > 0.25). Based on available data, the familiarity among Spanish affective words was comparable (*p* > 0.12).

The absolute value of the difference in valence and arousal values between Spanish and Italian words was computed and only those words with a difference of less than 0.70 were selected. Mixed ANOVA evidenced no significant differences between languages (Italian, Spanish) for valence, arousal, and number of letters (*p* > 0.52). For frequency, it was not possible to make comparisons because it was calculated differently in the Italian and Spanish databases. Familiarity could also not be compared between words in both databases because not all words in the Spanish database had a value for this dimension.

### Design

Participants were assigned to one of five possible conditions according to encoding (collaborative, individual) and Recall 1 (collaborative with the same partner, with another partner, and individual). The first group (Ind–Ind) encoded and recalled individually, the second group (Ind–Col) encoded individually and recalled collaboratively, the third group (Col–Ind) encoded collaboratively and recalled individually, the fourth group (Col–Col_sam_) encoded and recalled both collaboratively with the same partner and the fifth group (Col–Col_diff_) encoded collaboratively and recalled also collaboratively, but with a different partner (see Fig. 1).

### Procedure

We adopted a modified version of the collaborative memory procedure (Congleton & Rajaram, 2011, 2014; Pepe et al., 2021), designed to include three principal phases: Encoding (individual vs collaborative); Recall 1 (individual vs collaborative) and Recall 2 (individual). The whole session lasted approximately 60–75 min.

#### Encoding task: individual encoding condition

Participants sat individually at a desk. The experimenter informed the participants that the study aimed to examine memory and explained that 90 words, printed on index cards, would be presented scattered across the desk together with six cards containing 15 lines representing 6 very general categories (health, environment, education, news, relationships, and behavior). Participants were instructed to classify each word in one of the six possible categories. For each category, 15 words had to be selected so that at the end of the encoding task, no cards remained unclassified.

Participants were given 30 min to complete the task. If they finished categorizing the words before the time had elapsed, the experimenter instructed them to use the remaining time to revise the classification and make changes they felt necessary. A timer was placed on the desk indicating the time.

#### Encoding task: collaborative encoding condition

Instructions and procedures for the collaborative encoding task were identical to the individual encoding task, with the exception that two participants sat in a single desk where they both could see the words. The experimenter instructed participants to discuss and to come to an agreement about each individual word and the category in which to place it. Before beginning, both participants in each dyad were asked if they knew their partner, to ensure matching unknown people.

#### Distractor task 1

After the encoding task, participants completed a distractor task individually, where they spent 7 min playing a computer game similar to the Snake. Participants in collaborative groups moved to different rooms.

##### Recall 1

After the 7-min distractor period, participants completed a free recall task. Recall 1 could be individual, collaborative with the same or with a different partner from encoding, depending on the condition. They spent 7-min writing down all the words they could remember. In the collaborative group, one of the two participants was selected to list the words. Participants in the collaborative groups were instructed to reach a consensus about each item. In this manner, a word was listed only if they both agreed that it was present in the encoding task (see Harris et al., 2012, 2013). The experimenter instructed participants to keep trying to recall words until the time expired.

#### Distractor task 2

After Recall 1, participants completed a second distractor task individually practicing the computer game similar to Snake, for 5 min.

##### Recall 2

Following the distractor period, participants completed an individual free recall task identical to Recall 1. For seven minutes, participants in collaborative groups worked individually to recall as many words as possible. The experimenter instructed participants to keep trying to recall words until time expired.

#### Post-experimental debriefing

Finally, participants completed a post-experimental debriefing depending on which condition they were in. Participants in collaborative groups were asked whether their group had used any strategies to remember together and how they felt in the collaborative encoding and/or recall tasks. Participants were given the opportunity to ask questions and were fully debriefed and thanked for their participation.

## Results

We conducted a 2 × 3 × 5 × 2 mixed ANOVA with Recall (1 and 2) and valence (negative, neutral, positive) as within-subject factors, and condition (Ind–Ind, Ind–Col, Col–Ind, Col–Col_sam_, and Col–Col_diff_) and sample (Italian, Spanish) as between factors was conducted on recall measures (nominal and normal). Analogous ANOVAs without the valence variable were performed for the measures of ARC and intrusions.

A series of four orthogonal contrasts were used to test the study hypotheses. Specifically, the first contrast, which tests the first hypothesis, captures the collaborative encoding deficit by comparing the average of groups with individual encoding to the average of groups with collaborative encoding. The second contrast tests the classic collaborative inhibition effect (second hypothesis) by comparing the group Ind–Ind to the group Ind–Col. The third contrast tests the third hypothesis that predicts a reduction or elimination of the collaborative inhibition effect when recalling with the same partner (group Col–Ind vs group Col–Col_sam_). The fourth contrast, aimed to test the corresponding hypothesis, focuses on the groups that encoded collaboratively by comparing the group that encoded and recalled collaboratively with other partner with the average of the groups that recalled individually or collaboratively with the same encoding partner (group Col–Ind & group Col–Col_sam_ vs group Col–Col_diff_). This would allow us to determine whether recalling with another partner is more harmful than recalling with the same encoding partner or recalling individually. All *p* values reported in the present study were adjusted using the false discovery rate (FDR) correction for multiple comparisons at the 5% level (Benjamini & Yekutieli, 2001).

Table 1 includes descriptive statistics for all the measures on each condition. Analyses of nominal recall and ARC are presented below. Analyses on normal recall and intrusions are provided as supplementary material. Although repetitions were registered, their low number prevented us from analyzing them.

**Table 1:** Percentage of recalled words, ARC, intrusions, and repetitions for nominal and collaborative groups in the individual and collaborative conditions during Recall 1, 2

Recall condition	Encoding task	Recall group		Nominal recall	Percentage of recalled words	ARC	Percentage of intrusions	Percentage of repetitions
Recall 1	Individual	Individual	Ind–Ind	56.0 (10.50)	36.04 (12)	0.35 (0.27)	2.25 (2.36)	0.36 (0.63)
		Collaborative	Ind–Col	47.26 (9.0)	47.26 (8.91)	0.25 (0.17)	1.94 (1.73)	0.25 (0.62)
	Collaborative	Individual	Col–Ind	49.06 (11)	32.17 (11.13)	0.30 (0.20)	1.42 (1.57)	0.22 (0.57)
		Collaborative with the same partner	Col–Col_sam_	41.66 (11.40)	41.66 (11.40)	0.28 (0.26)	2.25 (1.61)	0.34 (0.70)
		Collaborative with different partner	Col–Col_diff_	39.30 (8.26)	39.30 (8.26)	0.17 (0.12)	1.47 (1.67)	0.31 (0.70)
Recall 2	Individual	Individual	Ind–Ind	56.11 (10.40)	36.32 (12.70)	0.41 (0.25)	2.58 (2.23)	0.27 (0.70)
		Collaborative	Ind–Col	57.74 (13.63)	40.47 (13.70)	0.41 (0.23)	2.2 (2.16)	0.50 (1.0)
	Collaborative	Individual	Col–Ind	50 (11)	32.64 (11.84)	0.32 (0.23)	2.08 (1.85)	0.22 (0.45)
		Collaborative with the same partner	Col–Col_sam_	48.09 (13.65)	35.74 (13.10)	0.31 (0.22)	2.47 (2.21)	0.26 (0.60)
		Collaborative with different partner	Col–Col_diff_	47.22 (10.46)	32.15 (9.10)	0.25 (0.19)	1.95 (1.65)	0.22 (0.63)

Values for each variable are means, with standard deviations in parentheses

*ARC* adjusted ratio of clustering

### Nominal recall

For the Col–Ind and Ind–Ind conditions, dyads were created by pairing two subjects that recalled individually and that were on consecutive time slots in a participation schedule. Nominal scores were obtained by summing the non-redundant items recalled by each member of the dyad. Thus, each item correctly recalled, whether by one partner or both partners of the same dyad, is computed as a single correct recall response.

**Fig. 2:**
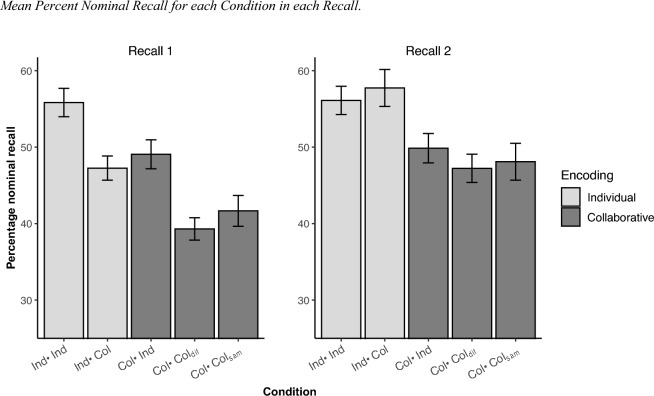
Mean Percent Nominal Recall for each Condition in each Recall. Note. Ind–Ind: individual encoding and individual recall, Ind–Col: individual encoding and collaborative recall, Col–Ind: collaborative encoding and individual recall, Col–Col_diff_, collaborative encoding and collaborative recall with a different partner, and Col–Col_sam_: collaborative encoding and collaborative recall with the same partner

Figure 2 shows the proportion of nominal recall in both Recall 1 and Recall 2 in each condition (see also Table 1). A 2 × 3 × 5 × 2 mixed ANOVA with Recall (1 and 2) and valence (negative, neutral, positive) as within-subject factors, and condition (Ind–Ind, Ind–Col, Col–Ind, Col–Col_sam_, and Col–Col_diff_) and sample (Italian, Spanish) as between factors was conducted on nominal recall percentage. The analysis yielded the main effects of Recall, *F*(1, 150) = 139.7, *p* < 0.001, *η*_*p*_^2^ = 0.482, and Condition, *F*(4, 150) = 7.72, *p* < 0.001, *η*_*p*_^2^ = 0.17, and the interactions Recall x Condition, *F*(4, 150) = 20.93, *p* < 0.001, *η*_*p*_^2^ = 0.358, Valence x Sample, *F*(2, 300) = 5.09, *p* = 0.019, *η*_*p*_^2^ = 0.033, and Recall × Valence, *F*(2, 300) = 4.48, *p* = 0.013, *η*_*p*_^2^ = 0.029. The data were collapsed across samples in subsequent analyses because neither sample (*F* < 1) nor the interaction Sample × Condition, *F*(2, 150) = 0.90, *p* = 0.654, *η*_*p*_^2^ = 0.023, proved to be significant.

In Recall 1, the contrasts showed collaborative encoding deficit and collaborative inhibition effects. Specifically, as predicted by our first hypothesis, a collaborative encoding deficit was found, given that groups that encoded individually showed a higher recall performance than the groups that encoded collaboratively, *t*(155) = 5.06, *p* = 0.001, *d* = 0.81*.* Consistent with the second hypothesis, the classic collaborative inhibition effect was observed. Among the groups that encoded individually, the group that recalled collaboratively had lower recall performance than the group that recalled individually, *t*(155) = 3.42, *p* = 0.003, *d* = 0.54; and an analogous collaborative inhibition effect was also found between the groups that encoded collaboratively, *t*(155) = 2.95, *p* = 0.011, *d* = 0.47, which is in line with the third hypothesis. Finally, among the groups that encoded collaboratively, the one that recalled collaboratively with a different partner showed lower recall performance than the other two groups (fourth hypothesis), *t*(155) = 2.79, *p* = 0.016, *d* = 0.45. Follow-up comparisons revealed that recall collaboratively with other partner resulted in lower recall performance than recall individually, *t*(155) = 3.89, *p* = 0.001, *d* = 0.624, but in similar performance than recall with the same partner, *t*(155) < 1.

Regarding Recall 2, only the collaborative encoding deficit was found (first hypothesis). Thus, the groups that encoded individually showed a higher recall performance than the groups that encoded collaboratively, *t*(155) = 4.44, *p* < 0.001, *d* = 0.71*.* All the other contrasts did not reach significance (*p*s > 0.1), indicating the absence of a collaborative inhibition effect.

#### ARC

**Fig. 3:**
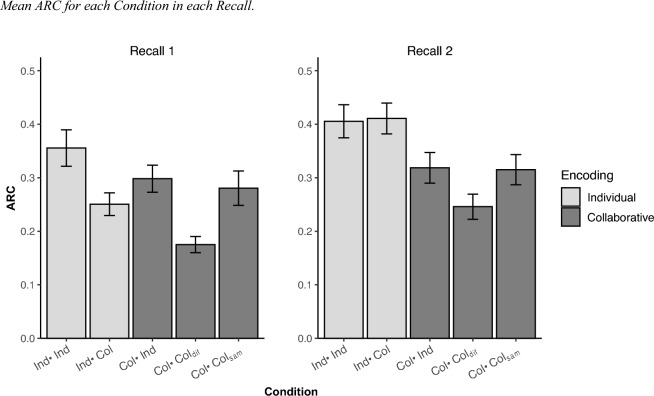
Mean ARC for each Condition in each Recall. *Note.* Ind–Ind: individual encoding and individual recall, Ind–Col: individual encoding and collaborative recall, Col–Ind: collaborative encoding and individual recall, Col–Col_diff_, collaborative encoding and collaborative recall with a different partner, and Col–Col_sam_: collaborative encoding and collaborative recall with the same partner

Figure 3 shows the mean ARC scores achieved in both Recall 1 and Recall 2 in each condition. An ARC value close to 1 shows that recall follows the category organization performed (i.e., perfect clustering), while an ARC value of 0 indicates a random recall pattern (Saraiva et al., 2017).

An overall 2 × 5 × 2 mixed ANOVA with recall (1 and 2) as within-subject factor and condition (Ind–Ind, Ind–Col, Col–Ind, Col–Col_sam_, Col–Col_diff_) and sample (Italian, Spanish) as between factors was conducted on ARC. The ARC value represents the proportion of actual category word repetitions to the total possible category repetitions. The analysis revealed the main effects of Recall, *F*(1, 310) = 35.07, *p* < 0.001, η_p_^2^ = 0.102, Condition, *F*(4, 310) = 6.57, *p* < 0.001, *η*_*p*_^2^ = 0.080, and the interaction Recall × Condition, *F*(4, 310) = 4.74, *p* = 0.004, *η*_*p*_^2^ = 0.058. Given that neither sample, *F*(1, 310) = 1.66, *p* = 0.346, *η*_*p*_^2^ = 0.005, nor the interaction Sample × Condition (*F* < 1) proved to be significant, the data were collapsed across samples in subsequent analyses.

Comparisons in Recall 1 did not reveal a collaborative encoding deficit, which is contrary to the first hypothesis, since there were no differences between groups that encoded individually and groups that encoded collaboratively, *t*(315) = 2.13, *p* = 0.08, *d* = 0.24. Consistent with the second hypothesis, the collaborative inhibition effect was found among the groups that encoded individually, as the group that recalled collaboratively had lower ARC than the group that recalled individually *t*(315) = 2.79, *p* = 0.016, *d* = 0.31*.* Interestingly, and in line with the third hypothesis, among the groups that encoded collaboratively, the collaborative inhibition effect was eliminated for the group that recalled collaboratively with the same partner as no differences were seen with the group that recalled individually, *t*(315) < *1.* Finally, and in agreement with the fourth hypothesis, among the groups that encoded collaboratively, the one that recalled collaboratively with other partner showed lower ARC than the other two groups, *t*(315) = 3.51, *p* = 0.002, *d* = 0.40. Follow-up comparisons revealed that the group that recalled with other partner resulted in lower ARC compared to both the group that recalled with the same partner, *t*(315) = 2.81, *p* = 0.016, *d* = 0.32, and the group that recalled individually, *t*(315) = 3.28, *p* = 0.004, *d* = 0.37. This suggests that recalling with a different partner hindered the level of organization in recall.

In Recall 2, a collaborative encoding deficit was observed (first hypothesis). Groups that encoded individually showed a higher ARC than groups that encoded collaboratively *t*(315) = 4.49, *p* < 0.001, *d* = 0.51*.* This result contrasts with what was found in Recall 1, where after applying the FDR correction the effect becomes not significant*.* Finally, among the groups that encoded collaboratively, there were no differences between the group that recalled collaboratively with other partner and the other two groups, *t*(315) = 2.06, *p* = 0.092, *d* = 0.23, which indicates that the collaborative inhibition effect does not persist over time.

## Discussion

To date, research on collaborative memory has mainly focused on the effects of collaboration in recall whereas studies on collaborative encoding are rare. In the present study, we investigated collaborative effects both at encoding and at recall by using a categorization task that stimulates discussion and active collaboration among participants (Basden et al., 1997, 2000) in two samples, one Italian and the other Spanish. Results showed collaborative encoding deficits and collaborative inhibition. We also found higher ARC scores in the conditions in which participants recalled alone or with the same encoding partner.

### Collaboration during encoding

We found a collaborative encoding deficit, whereby individuals who encoded together performed worse than individuals who encoded alone. This negative effect seems to be long-lasting since it remained both at Recall 1 and Recall 2 and occurred regardless of how recall was performed, as revealed by the absence of an interaction. Therefore, encoding deficits and collaborative inhibition seem to depend on different mechanisms. ARC scores were also lower in the collaborative than in the individual encoding condition, albeit only in the second recall, suggesting that the organization of the information is less integrated and more superficial when participants collaborate to classify the words. A possible explanation for the collaborative encoding deficit is that social interactions with other individuals divert attention away from the encoding task (Barber et al., 2012) and encourage divided attention (DA; Anderson et al., 1998; Baddeley et al., 1984; Craik et al., 1996). In our experiment, the individual encoding conditions allowed the participant to focus exclusively on the encoding task and the stimuli to be remembered, all of which may have favored subsequent recall.

Differently, in conditions involving collaborative encoding, the encoding of information while listening to the partner may be considered as a dual task that can be more or less demanding depending on the complexity of the information being communicated. Moreover, here participants not only have to attend to the memory task, but also to social information about the partner like their attitude, or facial expressions. They may also be more likely to be distracted or to focus their attention on aspects other than the information essential for successful task performance (e.g., discussions with a partner or explanation of why to place a word in a particular category). These additional aspects can be viewed as secondary tasks that compete for cognitive resources and potentially impact the primary task, resulting in an encoding deficit. These findings are in line with previous research from Pereira-Pasarin and Rajaram (2011) who argued that divided attention during encoding reduced retrieval organization in recall. In studies using various paradigms, DA during the study phase changed the quality of the encoding and shifted from a more semantically elaborated type to a shallow one (Naveh-Benjamin et al., 2000). In addition, DA negatively affects both memory and secondary task performance, compared to full attention (FA) conditions (Fernandes & Moscovitch, 2000; Naveh-Benjamin et al., 1998). Although research on collaborative memory has focused mainly on cognitive processes (see Weldon et al., 2000 for motivational factors), it could be relevant to determine whether other social processes (e.g., such as social conformity or fear of judgment from others) may mediate performance in collaborative groups.

### Collaboration during retrieval

Regarding the effects of collaboration during retrieval, we found the classic effect of collaborative inhibition, whereby dyads who recall individually have better nominal performance (Andersson et al., 2006; Marion & Thorley, 2016; Rajaram, 2011; Rajaram & Pereira-Pasarin, 2010; Saraiva et al. 2023). These results are also in line with findings from a previous study by Weldon and Balinger (1997) who observed that collaborative triads in the first recall evoked more information than individual subjects but less than nominal groups (the sum of the three non-redundant individual recalls). The effect of collaborative inhibition was also evident when encoding was collaborative. Indeed, the group that encoded collaboratively and recalled individually (Col–Ind group) remembered more information than the group that recalled dyadically with a different partner from the encoding one (Col–Col_diff_ group). This effect could be explicated through the retrieval disruption hypothesis (Basden et al., 1997), whereby the participant's retrieval strategy is interrupted by the partner's output. The pattern of the ARC scores, an index of organization in recall, is consistent with this explanation. Lower scores were observed in the Col–Col_diff_ than in the other groups, which suggests that only when the partners in the study and the recall phases were different the retrieval strategy of a participant was disrupted by the new partner.

Interestingly, the retrieval disruption hypothesis would also predict that the effect of collaborative inhibition should be eliminated or attenuated when participants organize information in a similar way (i.e., encoding with the same partner). However, quite unexpectedly, the negative effect on recall was present even when the participants in the dyad encoded the information together (Col–Col_sam_ group). It could be argued that the participants in this group did not use the organization of the information that they collaboratively generated; however, the analysis of the ARC scores indicates that recall output was compatible with the way in which the information was categorized. Specifically, there were no differences in ARC scores between groups that recalled individually or dyadically with the same partner (Col–Col_sam_ and Col–Ind groups). These findings contrast the results of Harris et al., (2013) who observed a collaborative inhibition elimination when the partners at study and at recall were the same. It is noteworthy, however, that Harris et al., (2013) included an individual recall phase before the recall in the different conditions. Thus, participants had already had a first individual recall in which the interruption of retrieval did not occur; perhaps the information after this first recall could become more consolidated and less susceptible to the negative effects of collaboration during the second (collaborative) recall.

The fact that participants in groups that collaborated during retrieval showed lower recall performance than those in groups recalling individually, regardless of the degree of organization of the output, suggests the involvement of additional factors in the collaborative inhibition effect. This could be explained by retrieval blocking or inhibition, a phenomenon for which remembering causes forgetting of other information in memory (Bäuml, 2008). Retrieval blocking occurring in collaborative recall conditions is thought to inhibit the recall of unquoted words by suppressing their memory representations and making them unavailable for retrieval (Anderson et al., 1994; Bäuml & Aslan, 2004). Moreover, in these conditions, part-set cuing, for which cuing is detrimental to memory performance, occurs due to blocking of retrieval, whereby cue words are believed to become stronger candidates for retrieval than noncue words; therefore, people in collaborative recall groups retrieve the reinforced cue words, blocking access to the unmentioned words (Rundus, 1973). It is also possible that an inhibitory mechanism as the social shared retrieval-induced forgetting plays a role (Coman & Hirst, 2015; Cuc et al., 2007). When individuals focus on a partner's recall of previously learned information, they may forget related information that the speaker did not mention.

An alternative explanation may be linked to the social contagion of memory errors (Basden et al., 2002; Davis & Meade, 2013; Huff et al., 2013; McNabb & Meade, 2014; Meade & Roediger, 2002; Roediger & McDermott, 1995; Roediger et al., 2001). For example, in collaborative encoding conditions, participants may incorporate their partner's erroneous suggestions into their own memories (Numbers et al., 2014). In the present experiment, participants in the group that collaborated in both the encoding and the recall phases (Col–Col_sam_) showed more intrusions in the first recall trial than the group that collaborated in the encoding phase but recalled individually (Col–Ind).It is possible that these participants incorporated each other’s errors during collaborative encoding and later, during collaborative recall, they were unable to correct each other, leading them to commit more errors.

Also in this case, as with collaboration during encoding, the DA hypothesis is applicable. Effectively the conditions where participants collaborate in retrieval may generate distraction and DA, unlike situations where recall is individual and thus FA is more feasible. DA is known to be deleterious to memory and secondary task performance, compared to FA conditions (Fernandes & Moscovitch, 2000; Naveh-Benjamin et al., 1998).

To recapitulate, collaboration during encoding and retrieval may lead to different effects driven by separate mechanisms. Specifically, the encoding deficit appears to be a consequence of inadequate learning of the material, potentially attributed to the divided attention demands in collaborative learning situations. On the other hand, the reduction of the collaborative inhibition effect arises, at least partially, from the similarity of retrieval strategies. Nevertheless, it is worth noting that this may not be the sole mechanism involved in the collaborative inhibition effect, given the distinct patterns observed for nominal recall and ARC measures. Consequently, it remains plausible that, in addition to retrieval disruption, other mechanisms such as inhibition, blocking, error contagion, and even divided attention during retrieval may also contribute to this effect.

The emotional valence of stimuli did not affect any of the effects described. Thus, both the collaboration deficit and the collaborative inhibition effects do not seem to be modulated by the valence of the material. An unexpected result was that the most remembered words were neutral words in comparison to both positive and negative words which in turn did not differ from each other. In general, increased memory performance has been repeatedly demonstrated for emotional material (Fairfield et al., 2013; Ferré et al., 2015; Talmi et al., 2013; Siddiqui & Unsworth, 2011; Zimmerman & Kelley, 2010), where negative or positive valanced stimuli are remembered better than neutral ones (Ke et al., 2017). A post hoc explanation for the pattern of results observed in the present study is that neutral words could be more concrete than negative and positive words and therefore they were better remembered (Yui et al., 2017).

Finally, the present study allowed us to confirm the generalizability of the results obtained with the new encoding task across two separate samples from different countries. Using the same paradigm with words from the same database adapted to two different languages (Bradley & Lang, 1999; Montefinese et al., 2014; Redondo et al., 2007), we found that memory performance was not affected by the different socio-cultural backgrounds of the different experimental samples. The study also confirmed the classic results of the collaborative memory paradigm such as collaborative encoding deficit, collaborative inhibition, and post-collaborative individual benefit.

The encoding task devised for the present study has proved to be useful for generating collaborative organization between participants. Nonetheless, the advantage of a shared organization may have been offset by the divided attention required to perform the task when collaborating with a partner. It could certainly be useful in the future to implement the encoding task in which the creation of a shared scheme of the encoded information is favored, but in a more controlled way, favoring focused attention. Regarding the emotional words used, certainly future studies should consider the concreteness of the words used as stimuli, to evaluate the emotive effect of the material.

## Conclusions

The present results add a new contribution to the study of collaborative memory, in particular regarding the effects of collaborative encoding, an aspect little explored in literature. To this end, we used five experimental conditions to investigate the possible differential effects on recall performance of individual versus collaborative encoding and retrieval. A further innovative aspect of this study was to examine whether the organization of the recall output was compatible with the organization established during encoding. Our results allowed us to confirm the generalizability of the results obtained with the new encoding task. In addition, we found that the classic collaborative inhibition effect was present even when the collaborative group showed a degree of recall output organization comparable to that of the individual recall group. We concluded that the detrimental effect of collaboration may not be entirely due to retrieval disruption, but rather may also be a result of divided attention.

### Supplementary Information

Below is the link to the electronic supplementary material.

Supplementary file1 (DOCX 25 KB)

## Data Availability

The datasets generated during and/or analyzed during the current study are available from the corresponding author on reasonable request.

## Footnotes

1 We have used the conventional term accepted in literature to refer to the collaborative inhibition effect (see review by Rajaram & Pereira-Pasarin, 2010). While this term might suggest an underlying inhibitory mechanism, here we use it at a descriptive level to denote a detrimental effect in recall.
