# Supplementary material for: Collaborative encoding with a new categorization task: a contribution to collaborative memory research
Source: Psychol Res. 2024 Mar 11;88(4):1339–51. doi: 10.1007/s00426-024-01929-w (PMC11142965; doi:10.1007/s00426-024-01929-w)
Supplement: Supplementary file 1 — Supplementary file1 (DOCX 25 KB) [file 426_2024_1929_MOESM1_ESM.docx]

**Supplementary Material**

**Normal Recall**

Normal recall is calculated by combining the recall responses of both partners without excluding redundant items (i.e., the same word recalled by two partners is computed as two correct responses). A preliminary 2 x 3 x 5 x 2 mixed ANOVA with recall (1 and 2) and valence (negative, neutral, positive) as within-subject factor, and condition (Ind-Ind, Ind-Col, Col-Ind, Col-Col_sam_, Col-Col_diff_) and sample (Italian, Spanish) as between factors was conducted on recall percentage. The analysis revealed the main effects of Recall, *F*(1, 310) = 97.0, *p* < .001, η_p_^2^ = .024, and Condition, *F*(4, 310) = 9.99, *p* < .001, η_p_^2^ = .114, and the interactions Recall x Condition, *F*(4, 310) = 19.75, *p* < .001, η_p_^2^ = .203, Valence x Sample, *F*(2, 620) = 6.80, *p* = .005, η_p_^2^ = .021, and Condition x Valence, *F*(8, 620) = 2.57, *p* = .027, *η_p_^2^* = .026. Given that neither sample (*F* < 1) nor the interaction Sample x Condition, *F(*4, 310) = 1.25, *p* = .45, η_p_^2^ = .016, proved to be significant, the data were collapsed across samples in subsequent analyses.

In Recall 1, contrasts showed a collaborative encoding deficit, and no collaborative inhibition effects. Specifically, groups that encoded individually showed a higher recall performance than the groups that encoded collaboratively, *t(315)* = 3.31, *p* = .004, *d* = 0.37*.* Among the groups that encoded individually, the group that recalled collaboratively had higher recall performance that the group that recalled individually, *t(315) =* 6.09, *p* < .001, *d* = 0.69*.* Among the groups that encoded collaboratively, the group that recalled collaboratively had higher recall performance that the group that recalled individually, *t(315)* = 5.16, *p* < .001, *d* = 0.58*.* Therefore, in line with previous research, normal recall of a collaborative group was greater than normal recall of any one individual (see Rajaram and Pereira-Pasarin, 2010)*.* Regarding Recall 2, only the encoding deficit was found, such that the groups that encoded individually showed a higher recall performance than the groups that encoded collaboratively, *t(315)* = 3.51, *p* = .002, *d* = 0.40*.* Other contrasts did not reach significance (*p* >.05), thus, failing to show the collaborative inhibition effect.

**Intrusions**

Table 1 in main text shows the number of intrusions in Recall 1 and Recall 2 for each condition. An overall 2 x 5 x 2 mixed ANOVA with recall (1 and 2) as within-subject factor and condition (Ind-Ind, Ind-Col, Col-Ind, Col-Col_sam_, Col-Col_diff_) and sample (Italian, Spanish) as between factors was conducted on intrusions (i.e., recalled items that did not appear on the original study list). The analysis yielded only a main effect of Recall, *F*(1, 310) = 14.95, *p* = .001, η_p_^2^ = .050. Given that neither sample, *F(*4, 310) = 1.85, *p* = .324, η_p_^2^ = .006, nor the interaction Sample x Condition (*F* < 1) proved to be significant, the data were collapsed across samples in subsequent analyses.

In Recall 1, the encoding deficit effect was not found since groups that encoded individually did not show different number of intrusions than groups that encoded collaboratively, *t(315)*  = 1.83, *p* =.14, *d* = 0.21*.* Between groups that encoded collaboratively, those that recalled individually showed fewer intrusions than those that recalled collaboratively with the same partner, *t(315)*  =2.58, *p* = .026, *d* = 0.29. As for Recall 2, no contrasts reached significance (*p*s > 0.4).
